# Supplementary material for: MVA Vector Vaccines Inhibit SARS CoV-2 Replication in Upper and Lower Respiratory Tracts of Transgenic Mice and Prevent Lethal Disease
Source: bioRxiv. 2021 Jan 1:2020.12.30.424878. Preprint. [Version 1] doi: 10.1101/2020.12.30.424878 (PMC7805450; doi:10.1101/2020.12.30.424878)
Supplement: Supplement 1 [file media-1.pdf]

MVA Vector Vaccines Inhibit SARS CoV-2 Replication in Upper and Lower Respiratory Tracts of Transgenic Mice and Prevent Lethal Disease

Ruikang Liu!, Jeffrey L. Americo!, Catherine A. Cotter, Patricia L. Earl, Noam Erez[1], Chen Peng[2] and Bernard Moss*

Laboratory of Viral Diseases, National Institute of Allergy and Infectious Diseases, National Institutes of Health, Bethesda MD 20892 USA

[!]Contributed equally

*Corresponding author: E-mail: bmoss@nih.gov

[1]Current address: Department of Infectious Diseases, Israel Institute of Biological Research (IIBR), Ness-Ziona, Israel

[2]Current address: China Agriculture University, Beijing, PRC

Supplemental Figure Legends

Fig. S1. Binding of hACE2. HeLa cells were infected with 5 PFU of rMVAs expressing WT or modified versions of S. After 24 h, the cells were incubated with soluble hACE2 and stained with Alexa Fluor 647-conjugated goat anti-hACE2 antibody. For the control, the addition of hACE2 protein was omitted. Histograms of two replicates are superimposed and M.F.I values of all infected cells are indicated.

Fig. S2. Time course of antibody production. C57BL/6 mice were vaccinated with MVA or rMVA *Tri*. Serum was collected before vaccination (Prebleeds) and 1 and 3 weeks after vaccination. **(A)** S-binding antibody determined by ELISA. **(B)** Neutralizing antibody determined by pseudovirus assay.

Fig. S3. Comparison of protein boosts. C57BL/6 mice were primed by IM injection with 10^7 PFU of rMVA *2P* into each hind leg. After 3 weeks, the mice were boosted by IM injection with 10 µg of RBD produced in human cells (h-RBD, Genscript), baculovirus produced RBD (RBD#1, Sino Biological), baculovirus produced RBD (RBD#2, provided by Eugene Valkov, NCI), or soluble S protein produced in human cells (h-S, Sino Biological). Each protein was administered with 15 µg of QS21 adjuvant. The mice were bled after 2 weeks and binding antibody and neutralization titers determined by ELISA **(A)** or pseudovirus assay **(B)**, respectively.

Fig. S4. MVA neutralizing antibody. Sera obtained from individual hACE2 mice that were primed with MVA *2P* (2PX1) and boosted with MVA *2P* (2PX2) or with RBD protein (2P/RBD

Pro) were tested for the ability to neutralize MVA using a flow cytometry assay. The dilutions of mouse sera that reduced the percentage of GFP-expressing cells by 50% (IC~~50~~) were plotted.

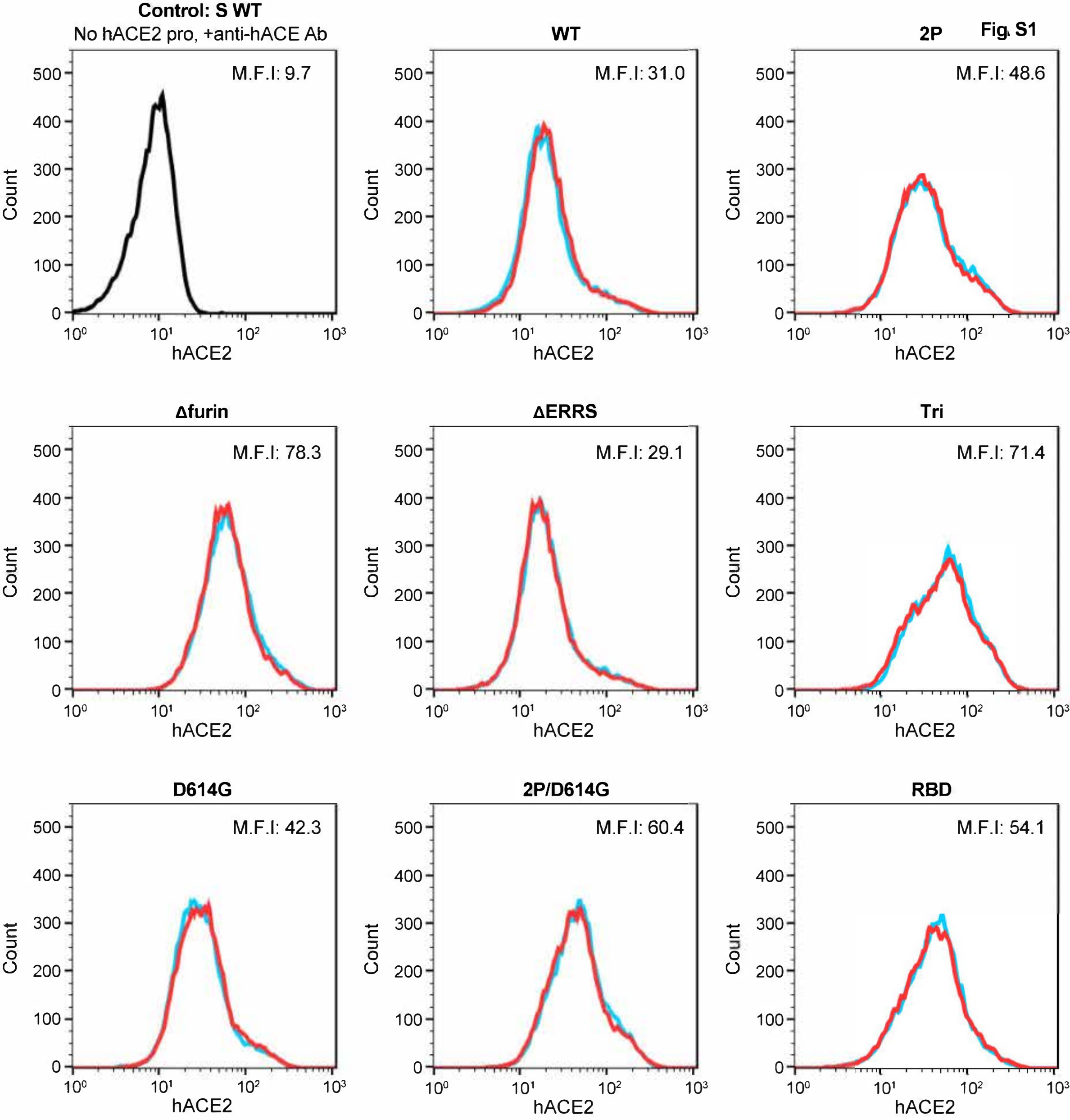
Fig. S1
Control: S WT
No hACE2 pro, +anti-hACE Ab
M.F.I: 9.7
WT
M.F.I: 31.0
2P
M.F.I: 48.6
Δfurin
M.F.I: 78.3
ΔERRS
M.F.I: 29.1
Tri
M.F.I: 71.4
D614G
M.F.I: 42.3
2P/D614G
M.F.I: 60.4
RBD
M.F.I: 54.1
Count
hACE2

Fig. S2

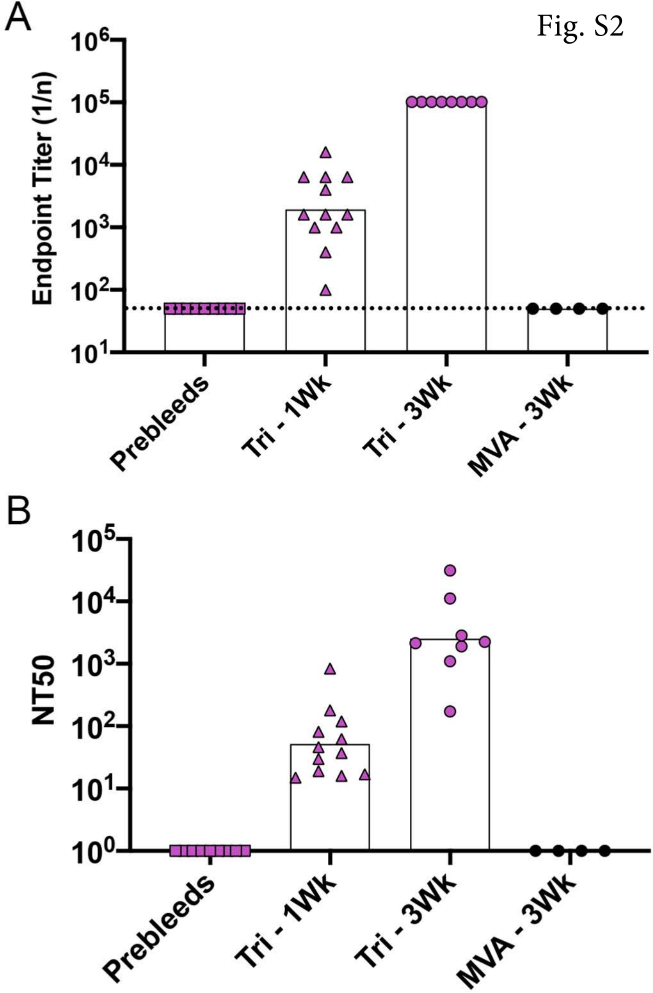

A
Endpoint Titer (1/n)
10^6
10^5
10^4
10^3
10^2
10^1
Prebleeds
Tri - 1Wk
Tri - 3Wk
MVA - 3Wk
B
NT50
10^5
10^4
10^3
10^2
10^1
10^0
Prebleeds
Tri - 1Wk
Tri - 3Wk
MVA - 3Wk

Fig. S3

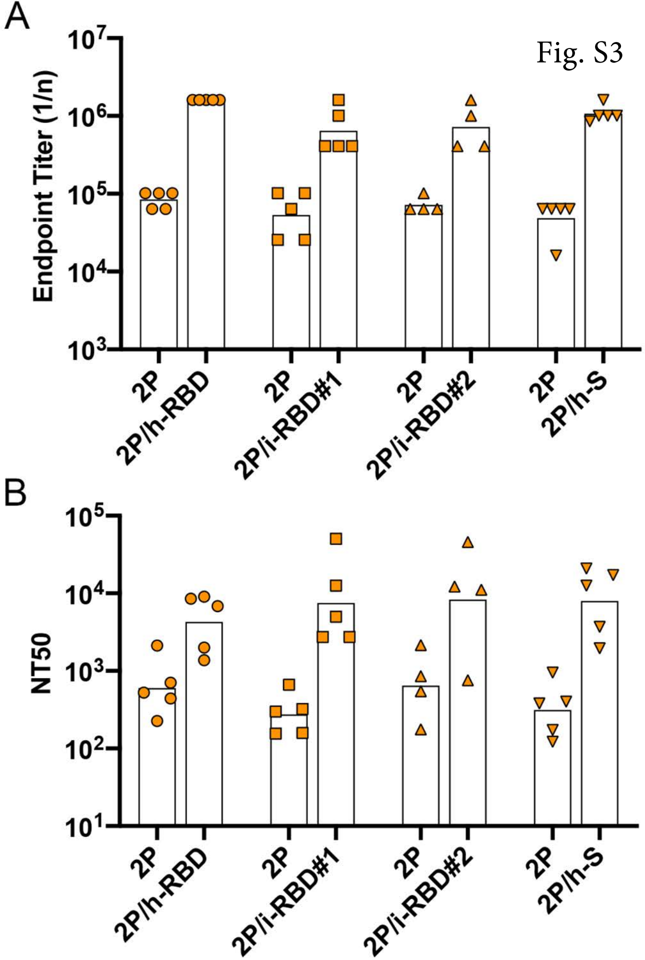
A
Endpoint Titer (1/n)
10^7
10^6
10^5
10^4
10^3
2P
2P/h-RBD
2P
2P/i-RBD#1
2P
2P/i-RBD#2
2P
2P/h-S
B
NT50
10^5
10^4
10^3
10^2
10^1
2P
2P/h-RBD
2P
2P/i-RBD#1
2P
2P/i-RBD#2
2P
2P/h-S

Fig. S4

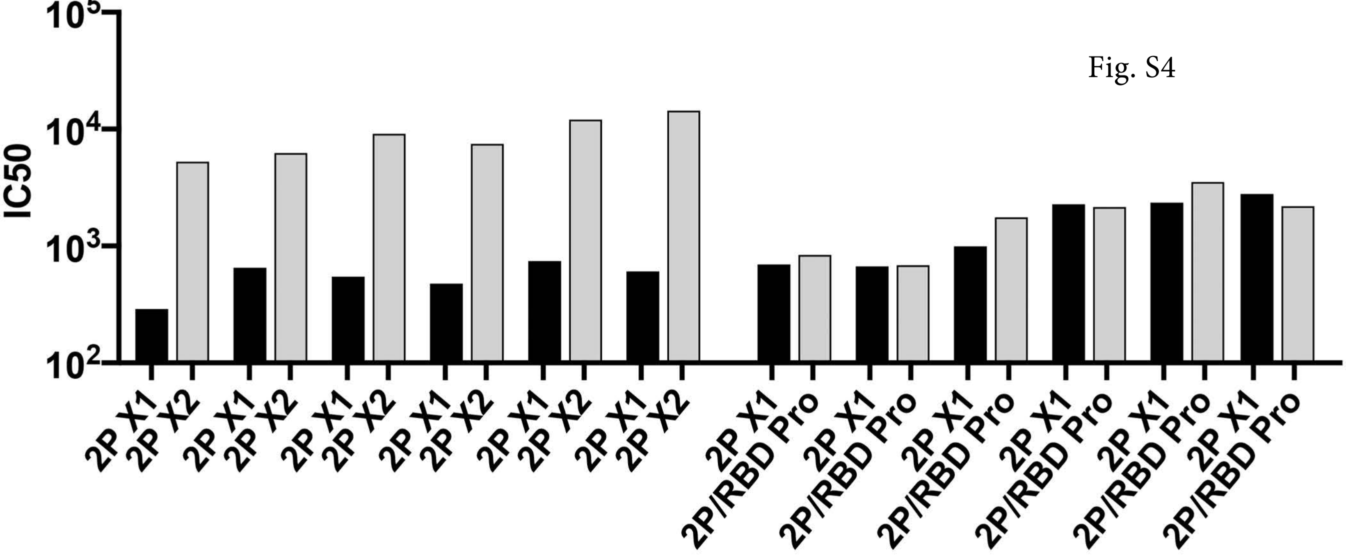
IC50
10^5
10^4
10^3
10^2
2P X1
2P X2
2P X1
2P X2
2P X1
2P X2
2P X1
2P X2
2P X1
2P X2
2P X1
2P X2
2P X1
2P/RBD Pro
2P X1
2P/RBD Pro
2P X1
2P/RBD Pro
2P X1
2P/RBD Pro
2P X1
2P/RBD Pro
2P X1
2P/RBD Pro
